# “Fly in the eye: Oestrus ovis” – a case report and a review from India

**DOI:** 10.3205/oc000254

**Published:** 2025-06-23

**Authors:** Richa Dhiman, Nancy Sharma, Ankita Sihag, Jasleen Singh

**Affiliations:** 1 Department of Ophthalmology, Maharishi Markandeshwar Institute of Medical Sciences and Research (MMIMSR), Mullana, India

**Keywords:** first instar larvae, ophthalmomyiasis extrerna, Oestrus ovis

## Abstract

Ophthalmomyiasis externa is the most common manifestation of *Oestrus ovis* (sheep nasal botfly) in humans. Several cases have been reported from various regions of India with the first case reported by Elliot in 1910. Here, we report such a case from North India along with the review of literature from India of the last fifteen years. A farmer presented to us with unilateral ocular symptoms of redness, foreign body sensation and severe watering in left eye who was misdiagnosed as acute conjunctivitis elsewhere. On slit lamp examination, multiple translucent larvae were found in his conjunctival sac. Microbiological analysis revealed them to be larvae of the *Oestrus ovis* fly. External ophthalmomyiasis is an uncommon entity with ocular symptoms mimicking acute conjunctivitis, hence a thorough examination in every suspicious case of acute red eye is important.

## Introduction

The word “myiasis” originated from the Greek word “myia”, which means fly. Ophthalmomyiasis refers to the infestation of orbit or ocular adnexa by larval forms of dipterous flies with *Oestrus ovis* being the most common. Based upon the part of the eye involved, they are broadly categorized as external, internal or orbital myiasis. It is predominantly seen in tropical areas, in people involved in animal husbandry and poor hygiene [1]. The disease is underreported and usually misdiagnosed as acute conjunctivitis or pre-septal cellulitis, which adds to morbidity. 

## Case description

**Figure 1:**
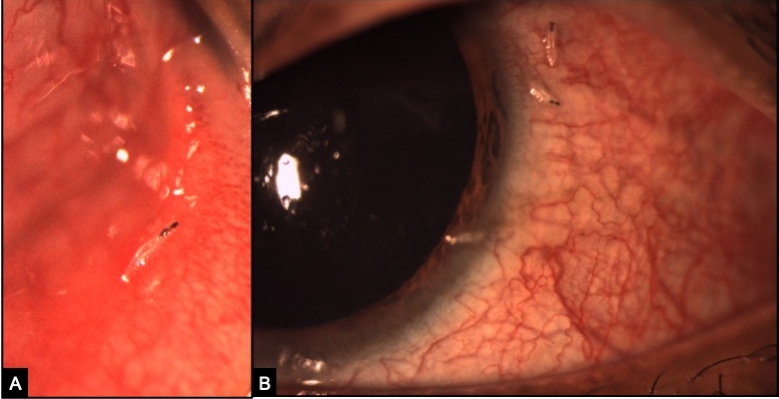
Slit lamp photo of the left eye showing (A) three larvae over the cornea, bulbar conjunctiva and (B) in palpebral conjunctiva with translucent segmented body and dark oral hooks.

**Figure 2:**
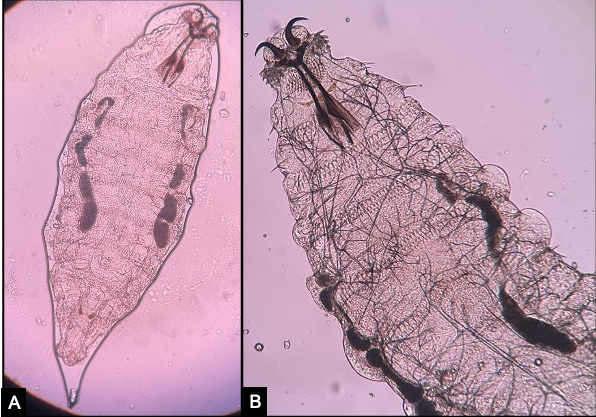
Microscopic morphology of *Oestrus ovis* showing (A) spindle-shaped larva with an eleven-segmented body and a pair of curved hooks in the anterior region. (B) Higher magnification focusing on the anterior end of larva depicting characteristic sharp, powerful dark brown oral hooks connected to the cephalopharyngeal skeleton.

A 23-year-old male, farmer by occupation, presented to us with a two-day history of foreign body sensation, redness, and excessive watering from his left eye. There was history of trauma by insect while working at sugarcane fields. Patient denies any history of livestock exposure. The past history was not significant for any ocular or medical problems. He was prescribed antibiotic eyedrops by a local practitioner making a diagnosis of acute conjunctivitis but his symptoms were not relieved. At presentation, his best corrected visual acuity (BCVA) was 20/20 in the right eye and 20/40 in the left eye. Examination of the right eye was normal while in the left eye there was a mild lid edema with conjunctival chemosis and superficial congestion. Extraocular movements were full. On slit-lamp examination, tiny, translucent larvae, 1–2 mm in size, with dark heads crawling over the bulbar conjunctiva and cornea were seen (Figure 1 (Fig. 1)). The larvae were photosensitive and moved towards the fornix on light exposure. Fundus examination was normal with no evidence of intraocular organism or inflammation. Using proparacaine 0.5% drops as topical anesthesia, 10 larvae were gently removed with fine, non-toothed forceps. Saline wet mount was made and the larvae were sent for microbiological examination. On examination, translucent white larvae of about 2 mm in length were noticed. Microscopy revealed a spindle-shaped skeleton with multiple segments and intersegmental spine bands (Figure 2 (Fig. 2)). A pair of sharp, dark brown oral hooks was attached to the internal cephalo-pharyngeal skeleton and tufts of numerous brown hooks were present on the anterior margins of each body segment. They were identified as the first-stage larvae of *Oestrus ovis*, the sheep nasal bot fly as per the entomological examination.

The patient was treated with topical steroids and antibiotics. On one week follow-up, the patient was completely relieved of his symptoms with normal anterior and posterior segment examination. 

## Discussion

*Oestrus ovis* belonging to the Diptera order and Oestridae family is a common parasite of sheep and goats. The adult female fly deposits its first instar larvae into the nostrils of the sheep or goat, where they develop into second and finally third instar larvae. A characteristic feature of *Oestrus ovis* is that it can deposit larvae without coming in direct contact with the host. It ejects a stream of larvae into the nostrils of the host and can deposit more than 500 eggs in the target area. Humans become the accidental host when these larvae are deposited on the ocular surface. These larvae do not secrete proteolytic enzymes and hence are usually confined to the outer membranes of the eye and do not develop beyond the first instar stage. They mostly die within ten days if not removed [2]. The characteristic feature of larvae of the *Oestrus ovis* is the spindle-shaped skeleton with dark brown curved oral hooks at the anterior end.

The first case of human ophthalmomyiasis in India was reported by Elliot in 1910 (Elliot RH 1968 quoted in [3]). Since then, multiple cases have been reported in the world, especially in tropical countries. The cases reported from India on *Oestrus ovis* infestation in last 15 years are summarized in Table 1 (Tab. 1). The youngest case reported was a 10-day-old child from Madhya Pradesh mimicking pre-septal cellulitis. All the cases had foreign body sensation with conjunctival congestion and watering. Only one case by Sreejith et al. reported the pseudo-membrane formation with keratitis [2]. Also, there was one case with bilateral ocular myiasis interna with posterior segment involvement where the patient presented with pre-septal cellulitis, uveitis, sub-retinal tracks and pre-retinal haemorrhages. Hence, over the last 15 years, the number of reported cases of ophthalmomyiasis by *Oestrus ovis* have increased in India with maximum cases reported from Madhya Pradesh.

This is the first reported case of ophthalmomyiasis externa by *Oestrus ovis* from Haryana in India. Our patient presented in April (summer season) with the features of unilateral redness, foreign body sensation, lacrimation as seen in the majority of cases reported from India. These clinical features can be easily mistaken for pre-septal cellulitis or acute conjunctivitis. It is therefore imperative to diagnose early and promptly intervene in all the cases to prevent serious complications such as ulcerative keratitis, blurring of vision and intraocular penetration causing panuveitis and retinal hemorrhages, which have been reported in the past. 

The management involves the removal of these larvae after putting anaesthetic drops to immobilize the larvae. Removal should be done with blunt tipped forceps as they tightly cling to the conjunctival surface and hence removal with a cotton tip applicator will not be successful. Removal followed by a week’s prescription of topical antibiotics and steroid usually results in successful treatment. However, a careful search for larvae by double everting the lids and a detailed posterior segment examination becomes necessary in some cases with strong clinical suspicion as rarely it can be found in the vitreous cavity or cause subretinal tracts with pre-retinal haemorrhage as reported in the literature. A recent travel history to endemic areas should raise a high index of suspicion for such situations. Oral ivermectin and oral steroids will be required to control the severe inflammation if larvae penetrate the posterior segment.

## Conclusion

With growing awareness of the ocular myiasis, an increase in number of such cases has been noted in the last five years in India. Our case serves as an addition in the growing literature about its diagnosis and management. 

### Take home points

- Ocular myiasis is considered an occupational disease among farmers and shepherds, but a high index of suspicion must be kept for anyone who presents with symptoms similar to unilateral acute conjunctivitis or pre-septal cellulitis.
- Cases usually increase during the early rainy season and summers.
- Patients with a recent travel history to endemic areas are at higher risk.
- A thorough slit lamp examination by an ophthalmologist can lead to prompt diagnosis thus preventing untoward complications.

## Notes

### Informed consent

Written informed consent was obtained for identifiable health information included in this case report.

### Acknowledgement

The authors would like to thank Dr. Jyoti Chauhan, Assistant Professor, Department of Microbiology, Maharishi Markandeshwar Institute of Medical Sciences and Research (MMIMSR), for confirming the identification of the larvae. 

### Author’s ORCID

- Richa Dhiman: 0000-0002-9284-0829

### Competing interests

The authors declare that they have no competing interests.

## Figures and Tables

**Table 1:**
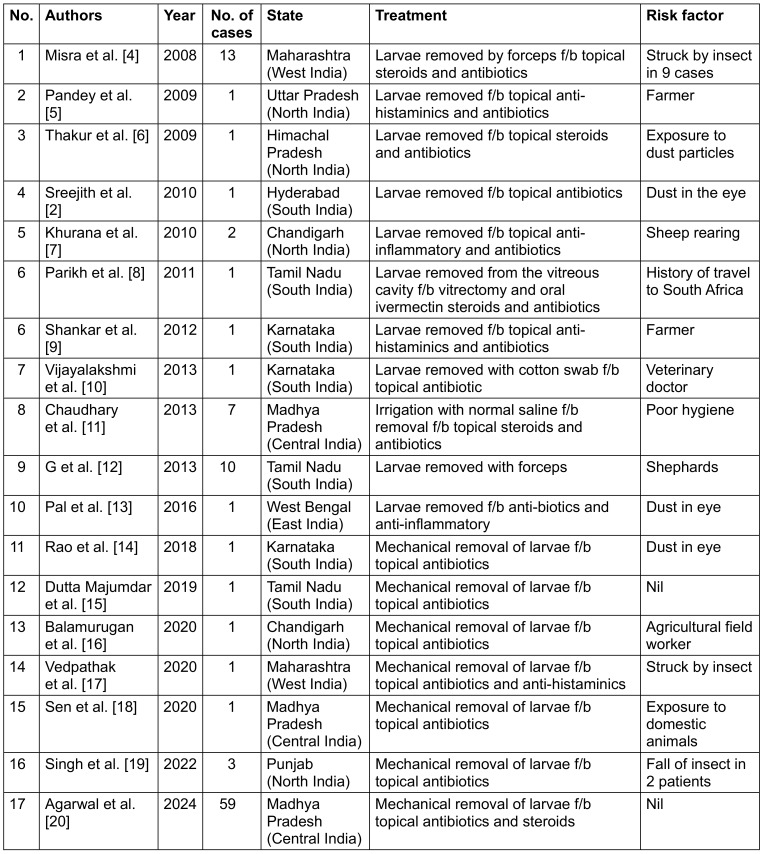
Reported cases of *Oestrus ovis* in the last 15 years from India
